# A Randomized Trial of Physical Therapy for Meniscal Tear and Knee Pain

**DOI:** 10.1056/NEJMoa2503385

**Published:** 2025-10-30

**Authors:** Jeffrey N. Katz, Jamie E. Collins, Leslie Bisson, Morgan H. Jones, James J. Irrgang, Faith Selzer, Clare E. Safran-Norton, Kurt P. Spindler, Heidi Y. Yang, Swastina Shrestha, Kim L. Bennell, James K. Sullivan, Melissa A. Kluczynski, Kaetlyn Arant, Maame Opare Addo, Jamie L. Huizinga, Zoe Zimmerman, Derek Sople, Peter Tonsoline, Madhuri Kale, William M. Wind, Antonia F. Chen, Michael Freitas, Bryson Lesniak, Kelly Jordan, Elizabeth G. Matzkin, Courtney Dawson, Lutul Farrow, Volker Musahl, John J. Leddy, Scott D. Martin, Elena Losina

**Affiliations:** 1 Brigham and Women’s Hospital, Harvard Medical School; 2 University at Buffalo Jacobs School of Medicine and Biomedical Sciences; 3 University of Pittsburgh Schools of Medicine and Health and Rehabilitation Sciences; 4 Cleveland Clinic and Lerner College of Medicine; 5 The University of Melbourne, Department of Physiotherapy; 6 University of Texas Southwestern Medical Center; 7 Massachusetts General Hospital

## Abstract

**Background:**

Physical therapy (PT) is routinely recommended for knee pain attributed to a degenerative meniscal tear, but its efficacy has not been established.

**Methods:**

We randomized participants 45–85 years old with knee pain, osteoarthritis, and meniscal tear to four arms: (1) Home Exercise (3-month home exercise program); (2) Home Exercise + text messages to encourage exercise adherence; Home Exercise + text messages + Standard PT (supervised strengthening, stretching, neuromuscular exercise, and manual therapy); and (4) Home Exercise + text messages + Sham PT (in-clinic sham manual therapy and sham ultrasound). The primary outcome was between-group difference in change in KOOS (Knee Osteoarthritis and injury Outcome Score) Pain scale (0 best to 100 worst) between baseline and 3-months, adjusting for site, baseline KOOS Pain, and radiographic grade.

**Results:**

We randomized 879 participants with mean age 59.2 (SD 7.8) years. The difference in three-month change between Home Exercise versus Home Exercise + text messages was −0.1 points (98.3% CI −3.8, 3.7), and Home Exercises versus Home Exercise + text messages + Standard PT was 2.5 points (98.3% CI −1.3, 6.2); the difference between Home Exercises + text messages versus Home Exercises + text messages + Standard PT was 2.5 points (98.3% CI −1.4, 6.5). Adverse events were rare, generally minor, and evenly distributed overall across arms.

**Conclusion:**

For patients with degenerative meniscal tear and knee pain, the addition of physical therapy or text messages to encourage adherence to home exercises was not superior in reducing pain to a home exercise program alone.

## Introduction:

Meniscal tear is present in 30-40% of middle-aged persons^1^ and ~ 80% of persons with osteoarthritis (OA).^2^ While it is uncertain whether the torn meniscus causes pain, the combination of degenerative meniscal tear and osteoarthritic change is frequently associated with pain, functional limitation, and resource utilization, including up to 400,000 arthroscopic partial meniscectomies annually in the US.^3^

Several randomized controlled trials (RCTs) reported that participants randomized to arthroscopic partial meniscectomy reported similar pain and function after one year compared to those randomized to in-clinic physical therapy (PT), home exercises, or both.^4-11^ Accordingly, treatment guidelines suggest PT or supervised exercise should be first-line treatments for knee pain and degenerative meniscal tear.^12-18^

It is unclear whether improvements following PT in these trials arose from physiological effects of exercises and/or interaction with physical therapists. TeMPO (Treatment of Meniscal Problems in Osteoarthritis) was a RCT designed to address whether adding text reminders to exercise or adding in-clinic PT result in greater pain relief than home exercises alone. It also addressed whether standard in-clinic PT is more effective than a sham regimen that provides comparable interaction with a therapist.

## Methods

### Participants

TeMPO is a four-arm RCT conducted at Brigham and Women’s Hospital, University at Buffalo, Cleveland Clinic, and University of Pittsburgh. Sites ceded oversight to the Mass General Brigham IRB. Eligible participants (Table S1) were 45-85 years old, with meniscal tear in the symptomatic compartment on MRI, and imaging evidence of an osteophyte or partial or full thickness cartilage damage in any compartment.^19^ The enrolling physician had to attest that the symptoms arose at least in part from meniscal tear in the symptomatic compartment. Participants with Kellgren-Lawrence (KL) 4 radiographs (advanced joint space loss) were ineligible.

### Recruitment and randomization

Research coordinators in each center pre-screened schedules of enrolling clinicians to identify potentially eligible patients.^19^ Eligible and interested participants underwent radiographs and MRI (if not obtained previously); those who remained eligible (Table S1) were randomized 1:1:1:1 to four arms in varying blocks of 4 and 8, stratified by site and KL grade (0-2 vs. 3). Personnel who assessed participants were blinded to treatment assignment.

### Study interventions

The study arms included: (1) Home Exercise (2) Home Exercise + Text Messages (3) Standard PT + Home Exercise + Text Messages (4) Sham PT + Home Exercise + Text Messages. The components of these interventions are detailed elsewhere^19,20^ and summarized below:

Home exercise involved stretching the quadriceps, hamstring, and gastrocnemius muscles and strengthening the gluteus maximus and medius, hamstring, and quadriceps muscles. Participants received an instructional pamphlet and video (on flash drive and the study website.) We provided ankle weights (1-pound increments from 0 to 10 pounds) and guidelines for progression of weights.^20^ In each arm except Home Exercise, participants received three text messages/week with theory-based statements encouraging exercise adherence.^21-23^ They also received pamphlets by mail twice-monthly for three months, which encouraged adherence.

For Standard PT, each session followed an unsupervised warm-up on an exercise bicycle and included: 1) manual therapy -- soft tissue and joint mobilization and stretching of tissues around the knee (5 minutes); and 2) therapist-directed strengthening and functional exercises, targeting the gluteus maximus and medius, hamstrings, and quadriceps muscles (25 minutes). Therapists could increase the intensity of exercises, switch one exercise for another, and modify the home program.^20^

Sham PT included elements not known to have physiologic benefit including 1) assessment of knee symptoms (5 minutes); 2) ultrasound of knee region with intensity set to 0 (12 minutes); 3) inert lotion applied gently along mid-thigh and distal tibia (5 minutes); and 3) sham manual therapy, consisting of minimal force to non-articular areas of the knee, without joint mobilization (8 minutes). Therapists did not ask about the home exercise regimen.

Participants receiving in-clinic PT were not told whether their regimen was intended to be sham or standard PT. Participants in each arm were instructed to do 100 minutes of exercise each week. In the Standard PT + Home Exercise + Text Messages arm the 100 minutes included home and in-clinic exercise. In the other arms, all 100 minutes were completed at home in four 25-minute sessions. Licensed physical therapists trained by the lead therapists at each center provided the Standard and Sham PT interventions. Visits were scheduled twice weekly in weeks 1-4, once weekly in weeks 5-8, once in week 10, and once in week 12 (total 14 sessions). During March-May 2020 we offered participants virtual PT visits because each site closed for COVID.

### Data sources and elements

Questionnaires completed at baseline and 3, 6, and 12 months included information on sex, weight, height, education, the Knee Injury and Osteoarthritis Outcome Score (KOOS) Pain and ADL (Activities of Daily Living) Scales (both scored 0-100, 100 = worst)^24^, and the EuroQol quality of life index (EQ-5D; range 0-100, 100 = perfect health).^25^

In musculoskeletal assessments conducted at baseline and three months, research coordinators blinded to arm assignment measured strength in the gluteus medius, quadriceps, and hamstring muscles using hand-held dynamometers. Participants performed the timed 40-meter walk, 30-second sit to stand, and single leg balance tests at baseline and three months.^26,27^

Participants were also asked to submit biweekly logs over the 12-week intervention period. One item asked how many days the participant completed their exercises in the prior week: 1, 2, 3, 4, or 5+.

### Outcome measures

The primary outcome was the change in KOOS Pain from baseline to 3 months.

We prespecified several secondary outcomes (Table S3). Among these was “failure,” defined as failing to improve by 8 points in KOOS Pain (a minimally clinically important change^28^) or receiving an intraarticular injection, arthroscopic partial meniscectomy, or total knee replacement, over this time period. Among participants not experiencing failure at 3 months, we defined treatment durability as maintaining at least an 8-point KOOS Pain improvement at 12-month follow-up while not receiving an intraarticular injection or knee surgery. Additional prespecified secondary outcomes included baseline to 3-month change in KOOS ADL, quadriceps, hamstring and gluteus medius strength, single-leg stand, 40-meter walk and timed sit-to-stand; and KOOS Pain, and KOOS ADL. We include EQ-5D as an exploratory outcome to illuminate broader impacts on quality of life.

### Adverse events

Serious adverse events included hospitalization, arthroscopic partial meniscectomy, and death. Adverse events included emergency department visits, ascertained from monthly medical record reviews, and musculoskeletal pain requiring an assistive device for at least one day, ascertained from biweekly logs.

### Statistical Analysis

#### Sample size

We powered TeMPO to detect an effect of 0.33 SD, which equates to 5.3 points on the KOOS Pain scale, given baseline SD of 16.^7^. Assuming 80% power and Type I error of 0.0167, each arm required 194 subjects. Allowing 10% dropout, we originally sought to enroll 214 subjects per arm. Because the dropout rate approached 13%, we increased the target to 220 per arm.

Based on the prespecified statistical analysis plan, we used linear regression with change in KOOS Pain as the primary outcome, adjusting for site, baseline KL grade (0-2 vs. 3), baseline KOOS Pain, and enrollment date (prior to or after March 15, 2020).^19^ We planned 3 primary comparisons: Home Exercise vs. Standard PT + Home Exercise + Text Messages; Home Exercise vs Home Exercise + Text Messages; and Home Exercise + Text Messages vs. Standard PT + Home Exercise + Text Messages. We used a Bonferroni-corrected p-value of 0.0167 for these 3 contrasts. Confidence intervals for secondary comparisons (which compared Sham PT + Home Exercise + text messages to the other three arms) have not been adjusted for multiplicity and should not be used for hypothesis testing.

We included a pre-specified sensitivity analysis excluding those enrolled between January and March 2020 (whose intervention periods overlapped with COVID shutdowns). We assessed the effect of treatment on binary failure with logistic regression, adjusting for baseline KOOS Pain, site, baseline KL grade, and enrollment date. In analyses of KOOS Pain, KOOS ADL, and EQ-5D at baseline, 3, 6, and 12 months, we used a linear mixed-effects model with unstructured covariance matrix, adjusted for site, KL grade (0-2 vs 3), and enrollment date. We performed an exploratory analysis stratified by radiographic grade (KL 0-2 vs KL3).

We examined adherence with the home exercise program with data from biweekly logs among participants who completed at least three of the six logs. We considered ≥3 days/week ‘adherent’ and calculated the mean proportion of adherent weeks across subjects in each arm.

We performed multiple imputation (MI) using chained equations, with imputation based on observed data (baseline KL grade, age, sex, BMI, baseline KOOS pain, baseline KOOS ADL, study site) and stratified by treatment group.^29^ We generated 20 imputed datasets for each outcome and combined data across imputations using Rubin’s rules.^30,31^ The primary analysis of 3-month change in KOOS pain assessed participants in the arms to which they were assigned, using data after MI. We also performed a complete case analysis restricted to subjects with baseline and 3-month data available. Additional analyses with longitudinal mixed-effects models utilized all available data, assuming missing data were missing at random.^32^ Details on analyses investigating robustness of the results to the missing data mechanism are provided in the Supplementary Appendix.

## Results

### Enrollment and baseline characteristics

Of 26,150 individuals screened between February 2017 and September 2022, we enrolled 1089, of whom 210 were subsequently excluded (Figure S1). We randomized 879 participants with mean age (SD) 59.2 (7.8) years, 67% KL grade 0-2, and baseline KOOS Pain 46.1 (15.5). The Buffalo and Boston sites enrolled 51% and 31% of participants, and the Pittsburgh and Cleveland sites 10% and 8%, respectively. Baseline features were similar across arms (Table 1). The sample included fewer Black (6%), Hispanic (4%), and Asian (2%) participants than the general US population (Table S2).

### Primary outcome

We did not observe meaningful differences in the three primary contrasts (Table 2). The difference in three-month change in KOOS Pain between the Standard PT + Home Exercise + Text Messages and the Home Exercise arms was 2.5 points (98.3% confidence interval (CI) −1.3, 6.2), as was the difference between Standard PT + Home Exercise + Text Messages and Home Exercises + Text Messages (2.5 points, 98.3% CI −1.4, 6.5). The difference between Home Exercises and Home Exercises + Text Messages was −0.1 (98.3% CI −3.8, 3.7).

Thirteen percent of participants dropped out by 3 months, 16% by 6 months, and 17% by 12 months. Dropout was similar between arms (Table S9). Results of analyses testing robustness of the “missing at random” assumption were similar to the primary analysis, as were results comparing complete case analyses to those using MI for missing data (Tables S10, S11).

### Secondary outcomes

At 3 months, the difference in change in KOOS Pain from baseline between Sham PT + Home Exercise + Text Messages and Standard PT + Home Exercise + Text Messages was 0.7 points (95% CI −3.7, 2.3). Binary treatment failure occurred at 3 months in 36% in the Home Exercise arm, 32% in Home Exercise + Text Messages, 30% in Sham PT+ Home Exercise + Text Messages, and 35% in Standard PT+ Home Exercise + Text Messages. Among 409 participants who did not experience treatment failure at 3-months, and for whom 12-month data were available, 77% met criteria for treatment durability in the Home Exercise arm, 81% in Home Exercise + Text Messages, 78% in Sham PT + Home Exercise + Text Messages, and 89% in Standard PT + Home Exercise + Text Messages. We did not observe meaningful differences across arms in KOOS Symptoms, KOOS ADL, and the strength and performance tests. Strength in the index knee increased similarly from baseline to three months across treatment arms (Table S4, S6).

At 6 months, the difference in KOOS Pain from baseline between the Standard PT + Home Exercise + Text Messages and Home Exercise arms was 4.1 (95% CI 0.7, 7.6). At 12 months, this difference was 2.5 points (95% CI −1.2, 6.2). KOOS Pain scores in the Standard PT + Home Exercise + Text Messages and Sham PT + Home Exercise + Text Messages were nearly identical at all timepoints. Longitudinal analysis of KOOS ADL and EQ-5D scores appeared consistent with findings for KOOS Pain (Table S7, Figures S2, S3).

### Adherence

Participants in the Sham PT + Home Exercise + Text Messages arm attended an average of 78% of the 14 visits scheduled compared with 77% in the Standard PT + Home Exercise + Text Messages arm. Results of the adherers analysis (participants who completed ≥ 8 in-person PT sessions) were similar to the primary analysis (Table S8). Sixty-nine percent of participants completed at least 3 home exercise logs. The mean proportion of weeks in which participants exercised at least three times was 77% for Home Exercise, 80% for Home Exercise + Text Messages, 82% for Sham PT + Home Exercise + Text Messages, and 76% for Standard PT+ Home Exercise + Text Messages.

We observed no meaningful differences in stratified analyses by site or KL grade (Tables S13, S14). The analysis excluding those enrolled between January and March 2020 yielded similar findings to the primary analysis.

### Adverse events

One participant (in Standard PT + Home Exercises + Text Messages) died and 33 (3.8%) had hospitalizations, including 14 (6.4%) in Home Exercise, 7 (3.2%) in Home Exercise + Text Messages, 5 (2.3%) in Sham PT + Home Exercise + Text Messages and 7 (3.2%) in Standard PT + Home Exercise + text messages (Table 3). Eighty subjects (9.1%) had arthroscopic partial meniscectomy on the index knee over 12 months, with similar percentages (8.2%-9.6%) in each arm. Emergency department visits for cardiovascular, neurological, pulmonary, and infectious reasons were rare and evenly distributed across arms (Table 3).

## Discussion

Whereas PT is recommended for persons with knee pain and degenerative meniscal tear,^12-18^ its efficacy in this setting has not been assessed rigorously. In TeMPO, participants randomized to home exercise alone; home exercise plus text messages to encourage adherence; and standard PT plus home exercise plus text messages, all improved in KOOS Pain by greater than one standard deviation between baseline and three months, with no clinically important or statistically significant differences between arms.

Participants assigned to in-clinic PT (Standard or Sham) had similar improvement in KOOS Pain between baseline and three months. The addition of in clinic PT (standard or sham) appeared to be associated with slightly greater pain improvement at 6 months compared to home exercises with no in-clinic PT. These findings emerged from secondary analyses without adjustment for multiplicity and should not be interpreted as definitive treatment effects. The proportion of participants adhering to home exercises during the first three months was virtually identical across all arms. Motivational text messages were not associated with differences in adherence to home exercises nor in pain outcomes.

Substantial evidence supports the efficacy of exercise for knee OA. ^33^ However, because all interventions included home exercises, we cannot determine whether the improvements observed in all study arms at three months were due to the home exercises or contextual factors such as attention and engagement attendant to participating in a trial, or regression to the mean.^34^

KOOS Pain scores in the Standard PT + Home Exercise + Text Messages and Sham PT + Home Exercise + Text Messages arms were virtually identical across all time points. These findings suggest that contextual effects are likely to explain the small apparent differences in pain between standard PT with home exercises versus home exercises alone over 12 months. Prior research has shown that 60-80% of the total effect of PT for knee OA can be attributed to contextual effects.^35^ While sham PT is not a true ‘placebo,’ our intent was to craft an intervention that controlled for interpersonal attention without having plausible biomechanical effects.

We note several limitations. Generalizability is limited by the small number of Black, Asian, and Hispanic participants (Tables S1, S2). While 30-minute PT visits mirror US practices, our findings should be generalized cautiously to settings with longer PT visits. More generally, our findings should not be extrapolated beyond the specific regimens investigated in TeMPO.

In conclusion, the combination of home exercises and physical therapy sessions did not result in greater pain reduction over three months than home exercises alone. Further, the addition of “motivational” text messages to home exercise did not improve pain outcomes over home exercise alone.

## Supplementary Material

Appendix

## Figures and Tables

**Figure 1:**
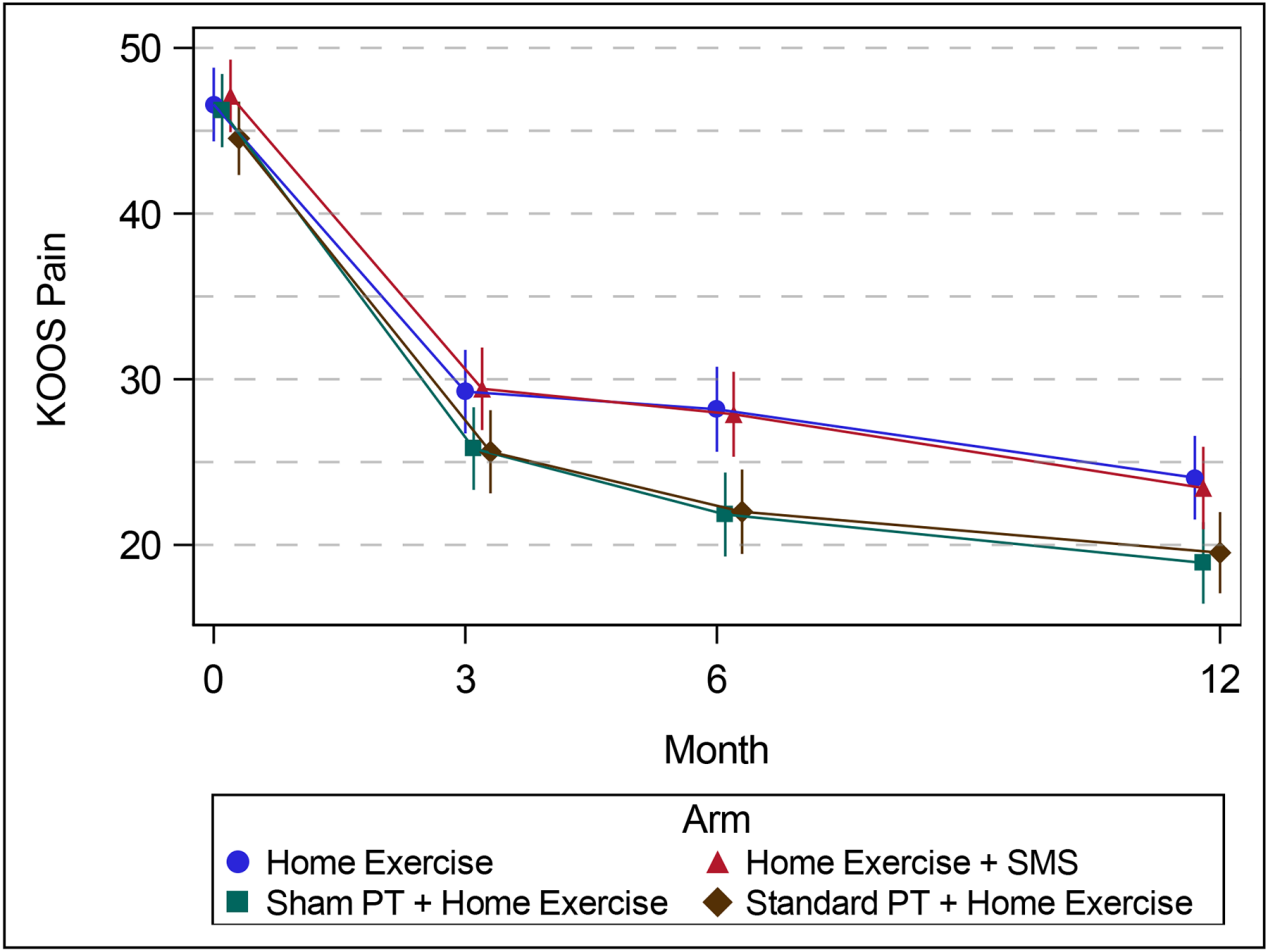
Adjusted* mean (95% CI)^ KOOS Pain over time across arms (0=no pain) from repeated measures models * Adjusted for site, KL grade (0-2 vs 3), and enrollment date ^Confidence intervals have not been adjusted for multiplicity and may not be used in place of hypothesis testing

**Table 1:** Baseline features of TeMPO study participants **according to** randomization arm

Characteristic	Home Exercise (N=218)	Home Exercise + Text Messages (N=222)	Sham PT + Home Exercise + Text Messages (N=220)	Standard PT + Home Exercise + Text Messages (N= 219)
**Sex**				
Female	114 (52%)	131 (59%)	132 (60%)	128 (58%)
Male	104 (48%)	91 (41%)	88 (40%)	91 (42%)
**Age** (mean, SD)	58.8 (8.1)	58.9 (7.5)	59.5 (7.5)	59.4 (8.1)
**BMI** (mean, SD)	30.1 (6.1)	29.7 (6.2)	30.2 (6.6)	30.2 (6.9)
**Race**				
White	180 (88%)	201 (92%)	192 (88%)	195 (90%)
Black	15 (7%)	12 (5%)	10 (5%)	11 (5%)
Asian	6 (3%)	2 (1%)	3 (1%)	4 (2%)
Other	3 (1%)	4 (2%)	12 (6%)	7 (3%)
**Ethnicity Hispanic or Latino**				
No	207 (97%)	213 (97%)	204 (94%)	209 (97%)
Yes	6 (3%)	7 (3%)	12 (6%)	6 (3%)
**Education**				
High school or less	17 (8%)	29 (13%)	27 (13%)	28 (13%)
More than high school	196 (92%)	193 (87%)	189 (88%)	189 (87%)
**KL Grade**				
0	24 (11%)	33 (15%)	33 (15%)	34 (16%)
1	102 (47%)	82 (37%)	93 (42%)	81 (37%)
2	20 (9%)	30 (14%)	23 (10%)	30 (14%)
3	72 (33%)	77 (35%)	71 (32%)	74 (34%)
**KOOS Pain** (mean, (SD) (0-100, 100 worst)	46.5 (15.7)	47.1 (14.3)	46.1 (16.1)	44.5 (16.1)
**Enrolled after March 15 2020**	85 (39%)	91 (41%)	89 (40%)	87 (40%)

1-2% of subjects missing data on race, ethnicity, education

KL Grade = Kellgren Lawrence radiographic grade

KOOS = Knee Osteoarthritis and injury Outcome Scale

**Table 2:** Primary Analysis: change in KOOS Pain from baseline to three months between pairs of treatment arms*

Primary Comparisons	Comparison Arms		ΔKOOS** Pain for A	ΔKOOS** Pain for B	Difference in ΔKOOS Pain (98.3% CI˄)		P-value
	A	B					
	Home Exercise	Home Exercise + Text Messages	−17.1	−17.0	−0.1 (−3.8, 3.7)		0.97
	Home Exercise	Standard PT+ Home Exercises + Text Messages	−17.1	−19.6	2.5 (−1.3, 6.2)		0.11
	Home Exercise + Text Messages	Standard PT + Home Exercises + Text Messages	−17.0	−19.6	2.5 (−1.4, 6.5)		0.12
Secondary Comparisons	Comparison Arms		ΔKOOS Pain for A	ΔKOOS Pain for B	Difference in ΔKOOS Pain (95% CI˄)		
	A	B					
	Home Exercise	Sham PT + Home Exercises + Text Messages	−17.1	−20.2	3.1 (0.1, 6.2)		
	Home Exercise + Text Messages	Sham PT + Home Exercises + Text Messages	−17.0	−20.2	3.2 (0.1, 6.3)		
	Sham PT + Home Exercises + Text Messages	Standard PT+ Home Exercises + Text Messages	−20.2	−19.6	−0.7 (−3.8, 2.5)		

* Adjusted for site, baseline KL grade, baseline KOOS Pain, COVID enrollment in multivariable linear regression

** Change in KOOS Pain within arm from baseline to three months

˄ 98.3% CI to account for three distinct primary comparisons; 95% CI for secondary comparisons. Confidence intervals for secondary comparisons have not been adjusted for multiplicity and may not be used in place of hypothesis testing.

**Table 3:** Adverse events by randomization arm*

Adverse Event	Home Exercise	Home Exercise +Text Messages	Sham PT + Home Exercise + Text Messages	Standard PT + Home Exercise + Text Messages
	N=218	N=222	N=220	N=219
Arthroscopic Partial Meniscectomy Index Knee	21 (9.6%)	21 (9.5%)	18 (8.2%)	20 (9.1%)
Death	0 (0%)	0 (0%)	0 (0%)	1 (0.5%)
Unplanned hospitalization	14 (6.4%)	7 (3.2%)	5 (2.3%)	7 (3.2%)
Total knee replacement	3 (1.4%)	2 (0.9%)	0 (0%)	1 (0.5%)
Knee pain (reported on exercise logs)**	8 (3.7%)	14 (6.3%)	16 (7.3%)	6 (2.7%)
Cardiovascular	1 (0.5%)	1 (0.5%)	2 (0.9%)	1 (0.5%)
Pulmonary	1 (0.5%)	0 (0%)	1 (0.5%)	0 (0%)
Infectious	3 (1.4%)	0 (0%)	0 (0%)	2 (0.9%)
Neurological	2 (0.9%)	1 (0.5%)	1 (0.5%)	1 (0.5%)
Other adverse event	10 (4.6%)	11 (5.0%)	10 (4.5%)	3 (1.4%)
Any adverse event	46 (21.1%)	48 (21.6%)	47 (21.4%)	35 (16.0%)

* The cell values represent the number of individuals with each event (a subject could have >1 event)

** knee pain resulting in use of walking aid for at least 24 hours
