# Supplementary material for: A Randomized Trial of Physical Therapy for Meniscal Tear and Knee Pain
Source: N Engl J Med. Author manuscript; Available in PMC 2025 Nov 13. (PMC12614259; doi:10.1056/NEJMoa2503385)
Supplement: Appendix [file NIHMS2098729-supplement-Appendix.pdf]

Randomized Trial of Physical Therapy for Meniscal Tear and Knee Pain (25-03385)

Supplementary Materials

Table of Contents

		Page
**List of investigators**		**3**
**Description of additional sensitivity analyses**		**4**
**Figures**		
S1	CONSORT diagram	**5**
S2	Adjusted Mean (95% CI) KOOS ADL over Time across Arms	**6**
S3	Adjusted Mean (95% CI) EQ5-D over Time across Arms	**7**
**Tables**		
S1	TeMPO Trial Inclusion and Exclusion Criteria	**8**
S2	Representativeness of Study Participants	**9**
S3	Primary and Secondary Outcome Measures in TeMPO Trial	**10**
S4	Primary and Secondary Continuous Outcomes at Baseline and 3 Months	**11**
S5	Adjusted Differences Between Randomization Arms in Change in Secondary Outcome Measures from Baseline to Three Months	**12-13**
S6	Adjusted Differences Between Randomization Arms in Change in Secondary Outcome Measures from Baseline to Three Months, Complete Case	**14-16**
S7	Adjusted Difference Between Pairs of Treatment Arms in Change in KOOS ADL from Baseline to 3, 6, and 12 Months in Repeated Measures Analysis	**17-18**
S8	Adjusted Differences Between Randomization Arms in Change in Secondary Outcome Measures from Baseline to Three Months, Adherers Analysis	**19-21**
S9	Data Completion by Timepoint, Outcome, and Arm	**22**
S10	Adjusted Differences Between Randomization Arms in Change in Secondary Outcome Measures from Baseline to Three Months, Multiple Imputation for Missing Data	**23-25**

S11	Adjusted* Differences Between Randomization Arms in Change in KOOS Pain from Baseline to Three Months, Sensitivity Analysis for Missing Data using Missing Not at Random Multiple Imputation	**26-27**
S12	Primary and Secondary 3-Month Outcomes by Site	**28**
S13	Adjusted 3-Month Outcomes by Site and Randomization Arm	**29-30**
S14	Adjusted 3-Month Outcomes by Site and KL Grade	**31-32**
S15	Adverse events in TeMPO Trial by Randomization Arm (Event Level)	**33**
**References**		**34**

TeMPO Investigators (all are authors)

Jeffrey N. Katz, MD, MSc[1]
Jamie E. Collins, PhD[1]
Leslie Bisson, MD[2]
Morgan H. Jones, MD, MPH[1]
James J. Irrgang, PhD, PT[3]
Faith Selzer, PhD[1]
Clare E. Safran-Norton, PT, PhD, MS[1]
Kurt P. Spindler, MD[4]
Heidi Y. Yang, MS, MPH[1]
Swastina Shrestha, MS[1]
Kim L. Bennell, PhD[5]
James K. Sullivan, MD, MS[1]
Melissa A. Kluczynski, MS[2]
Kaetlyn Arant, BA[1]
Maame Opare Addo, BA[1]
Jamie L. Huizinga, MD[1]
Zoe Zimmerman, BS[1]
Derek Sople, DPT[1]
Peter Tonsoline, PT[2]
Madhuri Kale, PT, DPT, MS[1]
William M. Wind, Jr., MD[2]
Antonia F. Chen, MD, MBA[6]
Michael Freitas, MD[2]
Bryson Lesniak, MD[3]
Kelly Jordan, PA[2]
Elizabeth G. Matzkin, MD[1]
Courtney Dawson, MD[1]
Lutul Farrow, MD[4]
Volker Musahl, MD[3]
John J. Leddy, MD[2]
Scott D. Martin, MD[7]
Elena Losina, PhD[1]

Brigham and Women's Hospital, Harvard Medical School; [1] University at Buffalo Jacobs School of Medicine and Biomedical Sciences; [2] University of Pittsburgh Schools of Medicine and Health and Rehabilitation Sciences; [3] Cleveland Clinic and Lerner College of Medicine; [4] The University of Melbourne, Department of Physiotherapy; [5] University of Texas Southwestern Medical Center; [6] Massachusetts General Hospital. [7]

Additional Sensitivity Analyses:

We performed several sensitivity analyses to address potential effects on the primary findings of study site, KL grade, and adherence to scheduled PT visits, as well as missing data mechanism. Results of these analyses are provided in Supplementary tables.

In a post-hoc analysis we examined primary and key secondary outcomes stratified by site, overall and by treatment arm, and stratified by KL grade (0-1, 2-3) and treatment arm. An additional adherers analysis excluded participants randomized to arms 3 or 4 who did not complete at least 8 in-person PT sessions.

For the primary outcome of 3-month change in KOOS Pain we performed additional sensitivity analysis for missing data, imputing missing data under a range of scenarios to test the robustness of study conclusions to potential not at random missingness. We imputed data assuming that missing data would be shifted towards the null (1) by 0.25 SD (2) by 0.5 SD (3) by 1 SD (4) by 0.5 SD for arms 1 and 2 only (5) by 0.5 SD for arms 3 and 4 only.

Figure S1: CONSORT diagram

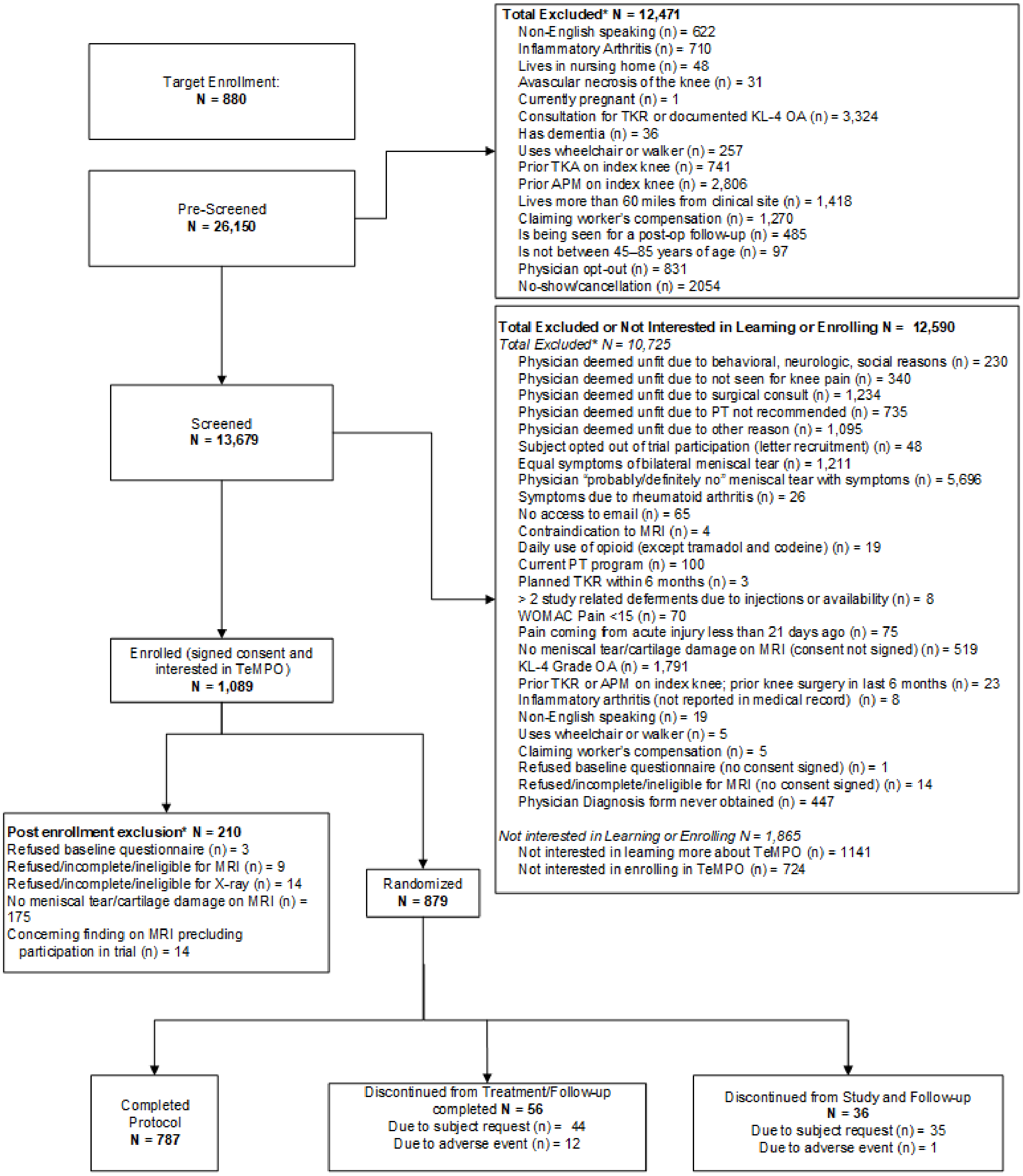

*Exclusions are not mutually exclusive

Figure S2. Adjusted* Mean (95% CI) KOOS ADL over Time across Arms**

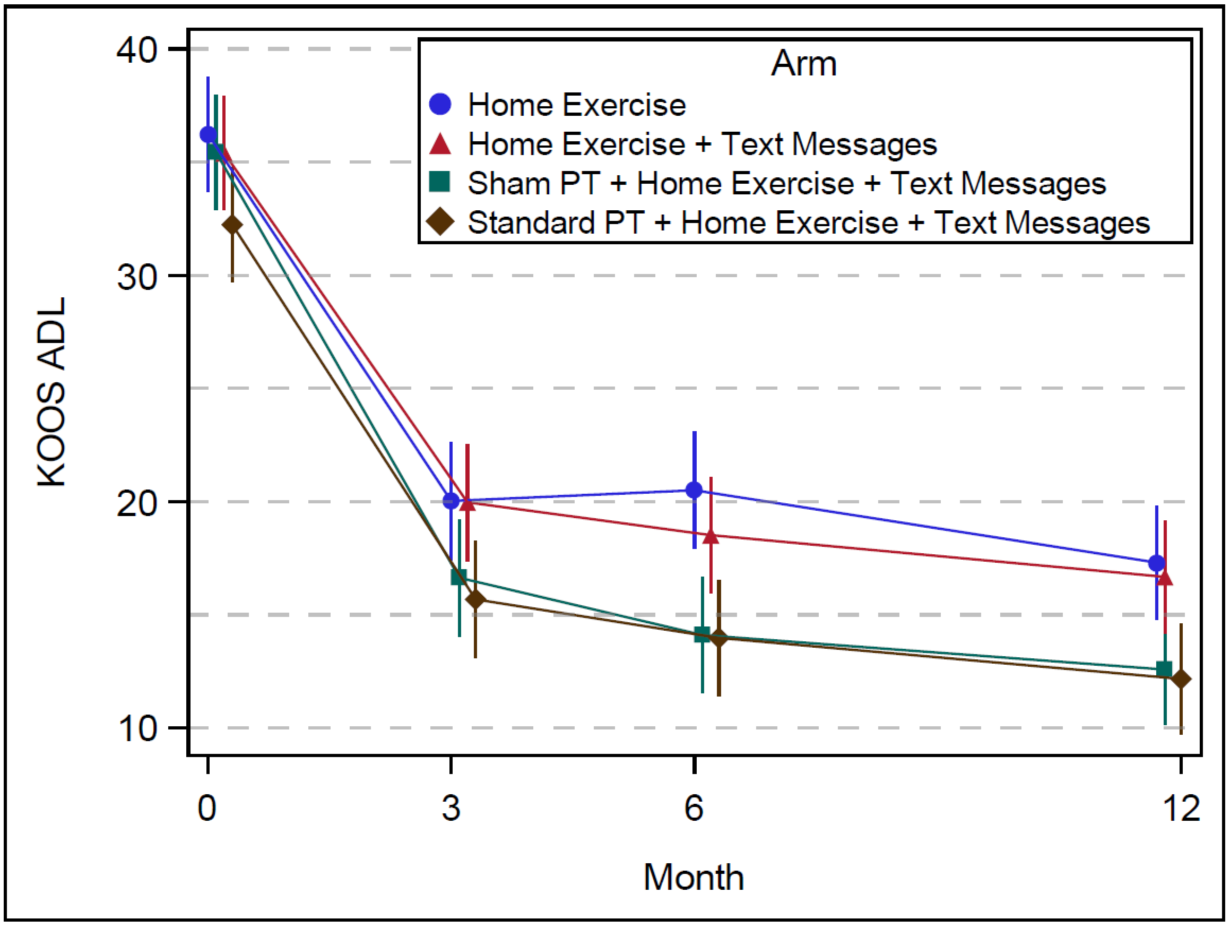

*Adjusted for site, KL grade (0-2 vs 3), and enrollment date

** Confidence intervals have not been adjusted for multiplicity and may not be used in place of hypothesis testing

Figure S3. Adjusted* Mean (95% CI) EQ5-D# over Time across Arms**

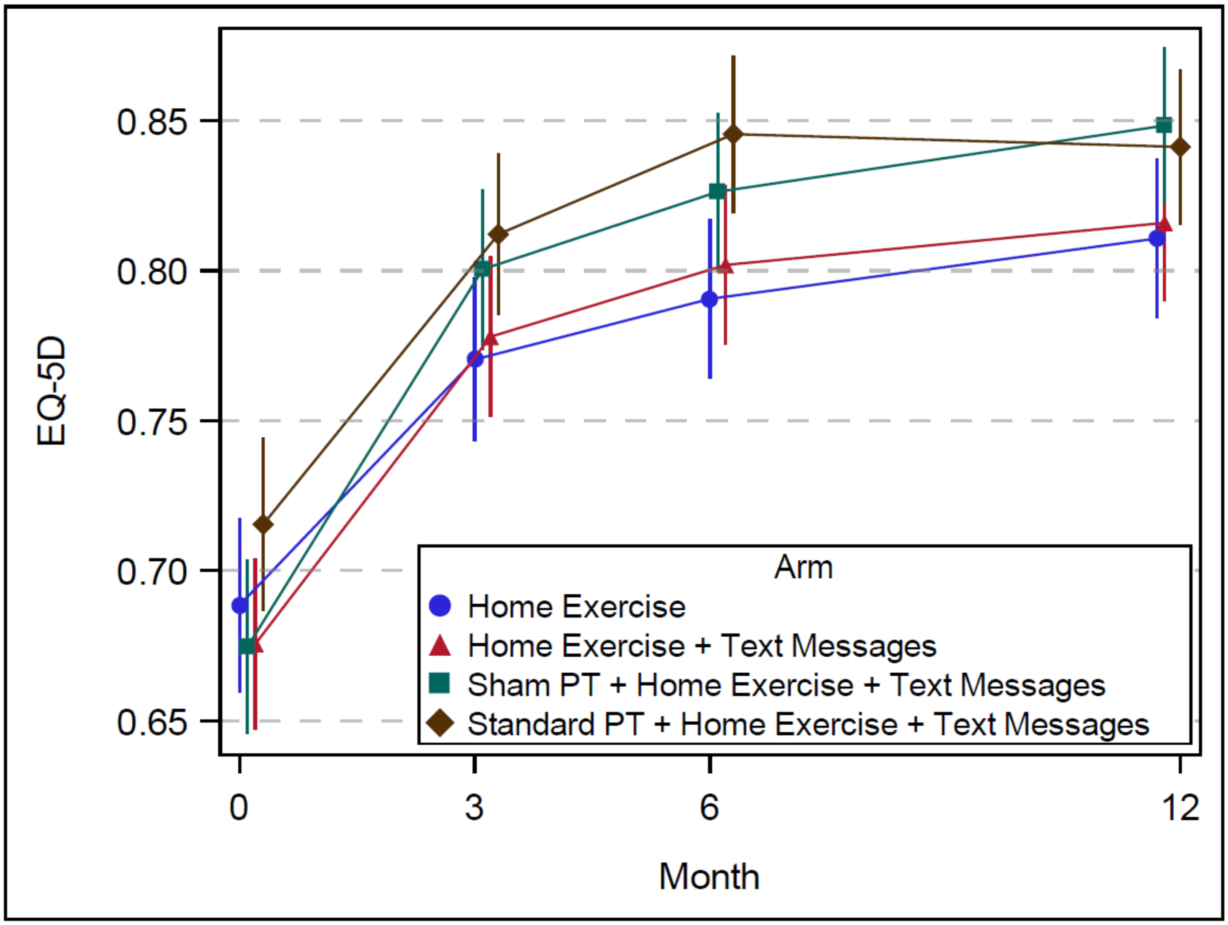

*Adjusted for site, KL grade (0-2 vs 3), and enrollment date
** Confidence intervals have not been adjusted for multiplicity and may not be used in place of hypothesis testing
#Analyses of EQ-5D are exploratory

Table S1: TeMPO Trial Inclusion and Exclusion Criteria

Inclusion Criteria	Exclusion Criteria
• Age 45–85<br>• Lives within 60 miles of a clinical site<br>• English speaking<br>• Has access to email<br>• Not living in a nursing home<br>• Physician suspects meniscal tear<br>• WOMAC* (knee) Pain score ≥15<br>• Meniscal tear present on MRI† (in last year), in symptomatic compartment<br>• Degenerative cartilage changes on MRI (taken in last year)	• Equal bilateral knee symptoms<br>• Clinician deems unfit for behavioral reasons<br>• Being seen for reason other than knee pain<br>• Contraindication to surgery, or physical therapy<br>• Pregnant<br>• Dementia documented in medical record<br>• Inflammatory arthritis, avascular necrosis of knee<br>• Surgery planned within 6 months on index knee<br>• Prior TKR or meniscal surgery on index knee<br>• Claiming worker's compensation for knee pain<br>• Requires use of wheelchair or walker<br>• Contraindication or concerning findings on MRI<br>• Clinician opposes unsupervised exercise<br>• Daily use of strong opioids<br>• Kellgren Lawrence grade 4 radiograph<br>• Currently in PT for knee for ≥ 2 weeks<br>• Knee pain arising from trauma <21 days ago

* Western Ontario and McMaster Universities Osteoarthritis Index
† Magnetic resonance imaging
‡ Total knee replacement

Table S2: Representativeness of Study Participants	
Condition under investigation	Knee pain in setting of meniscal tear in persons 45–85 years old
Considerations related to:	Comment
• Sex and gender	In this age group, meniscal tear is often associated with knee osteoarthritis, which is more common in women. As a result, in studies of knee pain with meniscal tear in this age group, women typically comprise 2/3 of the sample
• Age	This problem typically occurs in middle age and older persons, which is why most studies of this condition (including TeMPO) exclude younger individuals (< 45 in the case of TeMPO).
• Race or ethnic group	Underlying knee osteoarthritis is somewhat more prevalent in Blacks than Whites, Hispanics, and Asians. There is little evidence documenting the racial and ethnic composition of persons with knee pain and meniscal tear.
• Geography	There is little evidence documenting the prevalence of knee pain and meniscal tear in different countries.
• Other considerations	Treatment guidelines for persons with knee pain and meniscal tear are largely similar in the US, Europe, and Australia with an emphasis on initial management with physical therapy and /or strengthening and neuromuscular exercises.
• Overall representativeness of this trial	Subjects were asked on the baseline questionnaire to self-report their age in years, and to indicate whether their sex was female, male, or other. They were asked whether they considered themselves Hispanic and to self-report their race (White, Black, Asian, Native American, Native Hawaiian or Pacific Islander, other; they were permitted to check multiple categories.) With a mean age of 59 and female representation of 57%, the TeMPO sample closely resembles individuals treated for knee pain and meniscal tear across the US and globe. In the US, 14% of the population is Black, 7% Asian, and 19% is Hispanic. In TeMPO, 6% of the sample is Black, 2% Asian, 4% Hispanic. Each of these groups in underrepresented in the TeMPO sample.

**Table S3: Primary and Secondary Outcome Measures in TeMPO Trial**	
**Outcome Measure**	**Brief Description**
Primary Outcome	
KOOS Pain[1]	9 items aggregated unweighted; scored 0-100, 100 worse.
Secondary Outcomes	
Pain failure (binary)	Defined as improvement in KOOS Pain < 8 points, or receiving corticosteroid injection, arthroscopic partial meniscectomy, or total knee replacement
KOOS ADL[1]	17 items aggregated unweighted; scored 0-100, 100 worse
EQ-5D[24]	Quality of life index scaled 0-100 with 100=perfect health
Quadriceps Strength[3,4]	Best of three trials using handheld dynamometer; participant seated on examining table, knee flexed 90 degrees
Hamstring Strength[3,4]	Best of three trials using handheld dynamometer; participant seated on examining table, knee flexed 90 degrees
Gluteus Medius Strength[3,4]	Best of three trials using handheld dynamometer, participant lying on examining table on side, hips stacked
40-meter timed walk [5,6]	Four lengths between two cones spaced 10 meters apart
30-second timed up and go[5,6]	Using chair with no arm rest
Single leg stand[5,6]	Best of two trials

Table S4: Primary (KOOS Pain) and Secondary Continuous Outcomes at Baseline and 3 Months (mean (SD) unadjusted)				
**Outcome**	**Home Exercise**	**Home Exercise + Text Messages**	**Sham PT + Home Exercise + Text Messages**	**Standard PT + Home Exercise + Text Messages**
Baseline KOOS Pain (0-100, 100 worst)	46.5 (15.7)	47.1 (14.3)	46.1 (16.1)	44.5 (16.1)
M3 KOOS Pain (0-100, 100 worst)	28.3 (16.0)	29.1 (17.8)	25.5 (17.1)	25.3 (16.2)
**M3 KOOS Pain Change from Baseline**	**-17.4 (16.3)**	**-17.8 (17.3)**	**-20.4 (17.3)**	**-18.9 (16.1)**
Baseline KOOS ADL (0-100, 100 worst)	36.8 (17.7)	36.0 (16.6)	35.9 (18.6)	32.8 (18.8)
M3 KOOS ADL (0-100, 100 worst)	19.4 (17.5)	19.8 (18.2)	16.6 (17.9)	15.6 (15.3)
**M3 KOOS ADL Change from Baseline**	**-15.9 (17.8)**	**-15.2 (16.1)**	**-18.8 (17.8)**	**-16.1 (16.5)**
Baseline EQ-5D score	0.7 (0.2)	0.7 (0.2)	0.7 (0.2)	0.7 (0.2)
M3 EQ-5D score	0.8 (0.2)	0.8 (0.2)	0.8 (0.2)	0.8 (0.2)
**M3 EQ-5D Change from Baseline**	**0.1 (0.2)**	**0.1 (0.2)**	**0.1 (0.2)**	**0.1 (0.2)**
Baseline Quadriceps strength index knee (max of 3 trials)	50.3 (26.6)	50.8 (25.6)	48.6 (24.5)	48.8 (21.5)
M3 Quadriceps strength index knee (max of 3 trials)	58.4 (25.9)	58.5 (27.5)	56.7 (26.4)	56.4 (23.3)
**M3 Quadriceps strength index knee Change from Baseline**	**7.5 (15.9)**	**7.1 (13.1)**	**8.1 (13.9)**	**7.6 (14.9)**
Baseline Hamstrings strength index knee (max of 3 trials)	29.5 (14.7)	29.4 (14.0)	30.3 (14.9)	29.5 (13.8)
M3 Hamstrings strength index knee (max of 3 trials)	35.9 (16.0)	34.5 (14.9)	35.0 (15.5)	35.4 (13.7)
**M3 Hamstrings strength index knee Change from Baseline**	**5.6 (8.9)**	**4.5 (9.5)**	**5.0 (9.3)**	**6.0 (9.6)**
Baseline Gluteus Medius strength index knee (max of 3 trials)	36.1 (17.0)	35.3 (15.8)	34.8 (16.3)	35.0 (15.7)
M3 Gluteus Medius strength index knee (max of 3 trials)	41.7 (18.2)	41.1 (17.5)	39.7 (16.6)	40.9 (13.7)
**M3 Gluteus Medius strength index knee Change from Baseline**	**4.5 (10.3)**	**5.8 (9.8)**	**5.1 (10.7)**	**4.9 (9.7)**
Baseline Single Leg Balance index knee (max of 2 trials)	34.1 (22.7)	31.7 (22.1)	32.6 (21.5)	30.5 (22.4)
M3 Single Leg Balance index knee (max of 2 trials)	37.4 (22.6)	38.0 (21.7)	38.9 (22.0)	39.6 (21.9)
**M3 Single Leg Balance index knee Change from Baseline**	**3.7 (16.2)**	**6.8 (16.5)**	**7.0 (15.8)**	**6.9 (17.6)**
Baseline 40 m fast paced walk: Time(seconds):	27.0 (7.3)	27.5 (7.9)	27.3 (7.3)	27.4 (6.8)
M3 40 m fast paced walk: Time(seconds):	24.9 (6.2)	24.3 (5.8)	24.0 (4.8)	23.8 (4.4)
**M3 40 m fast paced walk: Time (secs) Change from Baseline**	**-1.8 (3.6)**	**-3.2 (5.4)**	**-3.0 (4.7)**	**-2.8 (4.9)**
Baseline 30 Second Sit to Stand: Number of Stands:	11.5 (3.9)	12.3 (4.2)	12.1 (4.5)	12.0 (4.4)
M3 30 Second Sit to Stand: Number of Stands:	13.9 (4.7)	15.6 (5.7)	14.9 (4.8)	14.9 (5.2)
**M3 30 Second Sit to Stand: # Stands Change from Baseline**	**2.1 (2.9)**	**3.3 (3.7)**	**3.0 (3.4)**	**2.8 (3.4)**

Table S5: Adjusted* Differences Between Randomization Arms in Change in Secondary Outcome Measures from Baseline to Three Months

Timepoint	A	B	ΔKOOS Pain for A	ΔKOOS Pain for B	Difference in ΔKOOS Pain (95% CI)^
**ΔKOOS Pain BL to 3 mo**	Home Exercise	Home Exercise + Text Messages	-17.3	-17.7	0.4 (-3.0, 3.7)
	Home Exercise	Sham PT + Home Exercises + Text Messages	-17.3	-20.4	3.1 (-0.2, 6.4)
	Home Exercise	Standard PT+ Home Exercises + Text Messages	-17.3	-18.9	1.6 (-1.7, 4.9)
	Home Exercise + Text Messages	Sham PT + Home Exercises + Text Messages	-17.7	-20.4	2.7 (-0.6, 6.0)
	Home Exercise + Text Messages	Standard PT+ Home Exercises + Text Messages	-17.7	-18.9	1.2 (-2.1, 4.6)
	Sham PT + Home Exercises + Text Messages	Standard PT+ Home Exercises + Text Messages	-20.4	-18.9	-1.5 (-4.8, 1.8)
**ΔKOOS Pain BL to 6 mo**	Home Exercise	Home Exercise + Text Messages	-18.4	-19.2	0.8 (-2.7, 4.3)
	Home Exercise	Sham PT + Home Exercises + Text Messages	-18.4	-24.4	6.0 (2.5, 9.5)
	Home Exercise	Standard PT+ Home Exercises + Text Messages	-18.4	-22.5	4.1 (0.7, 7.6)
	Home Exercise + Text Messages	Sham PT + Home Exercises + Text Messages	-19.2	-24.4	5.2 (1.7, 8.7)
	Home Exercise + Text Messages	Standard PT+ Home Exercises + Text Messages	-19.2	-22.5	3.3 (-0.2, 6.8)
	Sham PT + Home Exercises + Text Messages	Standard PT+ Home Exercises + Text Messages	-24.4	-22.5	-1.8 (-5.3, 1.6)
**ΔKOOS Pain BL to 12 mo**	Home Exercise	Home Exercise + Text Messages	-22.5	-23.7	1.1 (-2.6, 4.9)
	Home Exercise	Sham PT + Home Exercises + Text Messages	-22.5	-27.3	4.8 (1.1, 8.5)

	Home Exercise	Standard PT+ Home Exercises + Text Messages	-22.5	-25.0	2.5 (-1.2, 6.2)
	Home Exercise + Text Messages	Sham PT + Home Exercises + Text Messages	-23.7	-27.3	3.6 (0.0, 7.3)
	Home Exercise + Text Messages	Standard PT+ Home Exercises + Text Messages	-23.7	-25.0	1.3 (-2.3, 5.0)
	Sham PT + Home Exercises + Text Messages	Standard PT+ Home Exercises + Text Messages	-27.3	-25.0	-2.3 (-5.9, 1.4)
*Adjusted for site, baseline KL grade, baseline KOOS Pain, COVID enrollment in multivariable linear regression<br>^Confidence intervals for secondary comparisons have not been adjusted for multiplicity and may not be used in place of hypothesis testing.					

Table S6: Adjusted* Differences Between Randomization Arms in Change in Secondary Outcome Measures from Baseline to Three Months, Complete Case					
**Comparison Arms**		**ΔKOOS ADL for A**	**ΔKOOS ADL for B**	**Difference in ΔKOOS ADL**	**95% CI^**
**A**	**B**				
Home Exercise	Home Exercise + Text Messages	-15.6	-15.1	-0.5	-3.5, 2.5
Home Exercise	Standard PT + Home Exercise + Text Messages	-15.6	-17.5	1.9	-1.1, 4.9
Home Exercise + Text Messages	Standard PT + Home Exercise + Text Messages	-15.1	-17.5	2.4	-0.6, 5.4
Home Exercise	Sham PT + Home Exercise + Text Messages	-15.6	-18.4	2.8	-0.1, 5.8
Home Exercise + Text Messages	Sham PT + Home Exercise + Text Messages	-15.1	-18.4	3.3	0.4, 6.3
Sham PT + Home Exercise + Text Messages	Standard PT + Home Exercise + Text Messages	-18.4	-17.5	-0.9	-3.9, 2.0
**Comparison Arms**		**ΔEQ5-D# for A**	**ΔEQ5-D for B**	**Difference in ΔEQ5-D**	**95% CI^**
**A**	**B**				
Home Exercise	Home Exercise + Text Messages	0.084	0.097	-0.014	-0.044, 0.017
Home Exercise	Standard PT + Home Exercise + Text Messages	0.084	0.113	-0.030	-0.060, 0.001
Home Exercise + Text Messages	Standard PT + Home Exercise + Text Messages	0.097	0.113	-0.016	-0.056, 0.014
Home Exercise	Sham PT + Home Exercise + Text Messages	0.084	0.120	-0.036	-0.066, -0.006

Home Exercise + Text Messages	Sham PT + Home Exercise + Text Messages	0.097	0.120	-0.022	-0.052, 0.008
Sham PT + Home Exercise + Text Messages	Standard PT + Home Exercise + Text Messages	0.120	0.113	0.006	-0.024, 0.037
**Comparison Arms**		**ΔQuad Strength for A**	**ΔQuad Strength for B**	**Difference in ΔQuad Strength**	**95% CI^**
**A**	**B**				
Home Exercise	Home Exercise + Text Messages	7.2	7.0	0.2	-3.1, 3.5
Home Exercise	Standard PT + Home Exercise + Text Messages	7.2	7.0	0.1	-3.2, 3.4
Home Exercise + Text Messages	Standard PT + Home Exercise + Text Messages	7.0	7.0	-0.1	-3.3, 3.1
Home Exercise	Sham PT + Home Exercise + Text Messages	7.2	7.4	-0.3	-3.5, 3.0
Home Exercise + Text Messages	Sham PT + Home Exercise + Text Messages	7.0	7.4	-0.5	-3.6, 2.7
Sham PT + Home Exercise + Text Messages	Standard PT + Home Exercise + Text Messages	7.4	7.0	0.4	-2.8, 3.5
**Comparison Arms**		**ΔHam Strength for A**	**ΔHam Strength for B**	**Difference in ΔHam Strength**	**95% CI^**
**A**	**B**				
Home Exercise	Home Exercise + Text Messages	3.8	2.8	1.0	-1.0, 3.1
Home Exercise	Standard PT + Home Exercise + Text Messages	3.8	4.3	-0.5	-2.6, 1.5

Home Exercise + Text Messages	Standard PT + Home Exercise + Text Messages	2.8	4.3	-1.5	-3.5, 0.5
Home Exercise	Sham PT + Home Exercise + Text Messages	3.8	3.3	0.5	-1.5, 2.5
Home Exercise + Text Messages	Sham PT + Home Exercise + Text Messages	2.8	3.3	-0.5	-2.5, 1.4
Sham PT + Home Exercise + Text Messages	Standard PT + Home Exercise + Text Messages	3.3	4.3	-1.0	-3.0, 0.9

**Comparison Arms**		**Δgluteal strength for A**	**Δgluteal strength for B**	**Difference in Δgluteal strength**	**95% CI^**
**A**	**B**				
Home Exercise	Home Exercise + Text Messages	4.7	5.7	-1.0	-3.3, 1.3
Home Exercise	Standard PT + Home Exercise	4.7	4.9	-0.2	-2.5, 2.0
Home Exercise + Text Messages	Standard PT + Home Exercise + Text Messages	5.7	4.9	0.8	-1.5, 3.0
Home Exercise	Sham PT + Home Exercise + Text Messages	4.7	4.9	-0.2	-2.4, 2.1
Home Exercise + Text Messages	Sham PT + Home Exercise + Text Messages	5.7	4.9	0.8	-1.4, 3.0
Sham PT + Home Exercise + Text Messages	Standard PT + Home Exercise + Text Messages	4.9	4.9	-0.1	-2.2, 2.1

*Adjusted for site, baseline KL grade, baseline KOOS Pain, COVID enrollment in multivariable linear regression;

^Confidence intervals for secondary comparisons have not been adjusted for multiplicity and may not be used in place of hypothesis testing.

#EQ-5D analyses are exploratory

Table S7: Adjusted* Difference Between Pairs of Treatment Arms in Change in KOOS ADL and EQ-5D# from Baseline to 3, 6, and 12 Months in Repeated Measures Analysis

Comparisons		Pairwise difference in ΔKOOS ADL BL to 3 mo		Pairwise difference in ΔKOOS ADL BL to 6 mo		Pairwise difference in ΔKOOS ADL BL to 12 mo	
		**Estimate**	**95% CI^**	**Estimate**	**95% CI**	**Estimate**	**95% CI**
Home Exercise	Home Exercise + Text Messages	-0.8	-4.1, 2.6	1.2	-2.3, 4.6	-0.2	-3.9, 3.4
Home Exercise	Sham PT + Home Exercise + Text Messages	2.6	-0.8, 6.0	5.6	2.2, 9.0	3.9	0.3, 7.6
Home Exercise	Standard PT + Home Exercise + Text Messages	0.3	-3.0, 3.7	2.5	-0.9, 6.0	1.1	-2.5, 4.8
Home Exercise **+** Text Messages	Sham PT + Home Exercise + Text Messages	3.4	0.0,6.7	4.4	1.0, 7.9	4.1	0.5, 7.7
Home Exercise **+** Text Messages	Standard PT + Home Exercise + Text Messages	1.1	-2.3, 4.5	1.4	-2.1, 4.8	1.3	-2.3, 5.0
Sham PT + Home Exercise + Text Messages	Standard PT + Home Exercise + Text Messages	-2.2	-5.6,1.1	-3.1	-6.5, 0.4	-2.8	-6.4, 0.8
**Comparisons**		**Pairwise difference in ΔEQ-5D# BL to 3 mo**		**Pairwise difference in ΔEQ5-D BL to 6 mo**		**Pairwise difference in ΔEQ5-D BL to 12 mo**	
		**Estimate**	**95% CI**	**Estimate**	**95% CI**	**Estimate**	**95% CI**
Home Exercise	Home Exercise + Text Messages	-0.02	-0.06, 0.02	-0.02	-0.06, 0.01	-0.02	-0.06, 0.02
Home Exercise	Sham PT + Home Exercise +	-0.04	-0.08, -0.01	-0.05	-0.09, -0.01	-0.05	-0.09, -0.01

	Text Messages						
Home Exercise	Standard PT + Home Exercise + Text Messages	-0.01	-0.05, 0.02	-0.03	-0.07, 0.01	0.00	-0.04, 0.04
Home Exercise + Text Messages	Sham PT + Home Exercise + Text Messages	-0.02	-0.06, 0.01	-0.03	-0.06, 0.01	-0.03	-0.07, 0.01
Home Exercise + Text Messages	Standard PT + Home Exercise + Text Messages	0.01	-0.03, 0.04	0.00	-0.04, 0.03	0.01	-0.02, 0.05
Sham PT + Home Exercise + Text Messages	Standard PT + Home Exercise + Text Messages	0.03	-0.01, 0.06	0.02	-0.02, 0.06	0.05	0.01, 0.09

* Adjusted for site, baseline KL grade, baseline KOOS Pain, COVID enrollment
^Confidence intervals for secondary comparisons have not been adjusted for multiplicity and may not be used in place of hypothesis testing.
[#]EQ-5D analyses are exploratory

Table S8: Adjusted* differences between randomization arms in change in secondary outcome measures from baseline to three months, Adherers Analysis (excluded participants randomized to arms 3 or 4 who did not complete at least 8 in-person PT sessions)

**Comparison Arms**		**Δ KOOS Pain for A**	**Δ KOOS Pain for B**	**Difference in Δ KOOS Pain**	**95% CI^**
**A**	B				
Home Exercise	Home Exercise + Text Messages	-17.6	-17.4	-0.2	-3.2, 2.8
Home Exercise	Sham PT + Home Exercise + Text Messages	-17.6	-20.2	2.7	-0.4, 5.8
Home Exercise	Standard PT + Home Exercise + Text Messages	-17.4	-20.2	2.9	-0.2, 6.0
Home Exercise + Text Messages	Sham PT + Home Exercise + Text Messages	-17.6	-21.2	3.7	0.6, 6.7
Home Exercise + Text Messages	Standard PT + Home Exercise + Text Messages	-17.4	-21.2	3.9	0.8, 6.9
Sham PT + Home Exercise + Text Messages	Standard PT + Home Exercise + Text Messages	-21.2	-20.2	-1.0	-4.1, 2.2
**Comparison Arms**		**Δ KOOS ADL for A**	**Δ KOOS ADL for B**	**Difference in Δ KOOS ADL**	**95% CI**
**A**	B				
Home Exercise	Home Exercise + Text Messages	-15.7	-15.2	-0.5	-3.4, 2.4
Home Exercise	Sham PT + Home Exercise + Text Messages	-15.7	-18.0	2.3	-0.8, 5.3
Home Exercise	Standard PT + Home Exercise + Text Messages	-15.2	-18.0	2.8	-0.3, 5.8
Home Exercise + Text Messages	Sham PT + Home Exercise + Text Messages	-15.7	-19.5	3.8	0.8, 6.8
Home Exercise + Text Messages	Standard PT + Home Exercise + Text Messages	-15.2	-19.5	4.3	1.3, 7.3
Sham PT + Home Exercise + Text Messages	Standard PT + Home Exercise + Text Messages	-19.5	-18.0	-1.5	-4.6, 1.6
**Comparison Arms**					**95% CI**

A	B	Δ EQ5-D[#] for A	Δ EQ5-D for B	Difference in Δ EQ5-D	
Home Exercise	Home Exercise + Text Messages	0.08	0.10	-0.01	-0.04, 0.02
Home Exercise	Sham PT + Home Exercise + Text Messages	0.08	0.11	-0.03	-0.06, 0.00
Home Exercise	Standard PT + Home Exercise + Text Messages	0.10	0.11	-0.02	-0.05, 0.01
Home Exercise + Text Messages	Sham PT + Home Exercise + Text Messages	0.08	0.13	-0.05	-0.08, -0.02
Home Exercise + Text Messages	Standard PT + Home Exercise + Text Messages	0.10	0.13	-0.03	-0.06, 0.00
Sham PT + Home Exercise + Text Messages	Standard PT + Home Exercise + Text Messages	0.13	0.11	0.01	-0.02, 0.05
**Comparison Arms**		**Δ Quad Strength for A**	**Δ Quad Strength for B**	**Difference in Δ Quad Strength**	**95% CI**
**A**	**B**				
Home Exercise	Home Exercise + Text Messages	7.2	7.0	0.2	-3.1, 3.5
Home Exercise	Sham PT + Home Exercise + Text Messages	7.2	6.9	0.3	-3.1, 3.6
Home Exercise	Standard PT + Home Exercise + Text Messages	7.0	6.9	0.1	-3.2, 3.3
Home Exercise + Text Messages	Sham PT + Home Exercise + Text Messages	7.2	7.6	-0.4	-3.7, 2.9
Home Exercise + Text Messages	Standard PT + Home Exercise + Text Messages	7.0	7.6	-0.6	-3.8, 2.6
Sham PT + Home Exercise + Text Messages	Standard PT + Home Exercise + Text Messages	7.6	6.9	0.7	-2.6, 3.9
**Comparison Arms**		**Δ Hamstring Strength for A**	**Δ Hamstring Strength for B**	**Difference in Δ Hamstring Strength**	**95% CI**
**A**	**B**				

Home Exercise	Home Exercise + Text Messages	3.8	2.8	1.0	-1.0, 3.1
Home Exercise	Sham PT + Home Exercise + Text Messages	3.8	4.0	-0.2	-2.3, 1.9
Home Exercise	Standard PT + Home Exercise + Text Messages	2.8	4.0	-1.2	-3.2, 0.8
Home Exercise + Text Messages	Sham PT + Home Exercise + Text Messages	3.8	3.2	0.6	-1.5, 2.6
Home Exercise + Text Messages	Standard PT + Home Exercise + Text Messages	2.8	3.2	-0.4	-2.4, 1.6
Sham PT + Home Exercise + Text Messages	Standard PT + Home Exercise + Text Messages	3.2	4.0	-0.8	-2.8, 1.3
**Comparison Arms**		**Δ gluteal strength for A**	**Δ gluteal strength for B**	**Difference in Δ gluteal strength**	**95% CI**
**A**	**B**				
Home Exercise	Home Exercise + Text Messages	4.8	5.8	-1.0	-3.3, 1.3
Home Exercise	Sham PT + Home Exercise + Text Messages	4.8	4.8	-0.1	-2.4, 2.2
Home Exercise	Standard PT + Home Exercise + Text Messages	5.8	4.8	1.0	-1.3, 3.2
Home Exercise + Text Messages	Sham PT + Home Exercise + Text Messages	4.8	4.9	-0.2	-2.5, 2.1
Home Exercise + Text Messages	Standard PT + Home Exercise + Text Messages	5.8	4.9	0.8	-1.4, 3.1
Sham PT + Home Exercise + Text Messages	Standard PT + Home Exercise + Text Messages	4.9	4.8	0.1	-2.1, 2.4

* Adjusted for site, baseline KL grade, baseline KOOS Pain, COVID enrollment
^Confidence intervals for secondary comparisons have not been adjusted for multiplicity and may not be used in place of hypothesis testing.
#EQ-5D analyses are exploratory

Table S9: Data Completion by Timepoint, Outcome, and Arm					
**Timepont**	**Outcome**	**N (%)**			
		**Home Exercise**	**Home Exercise + Text Messages**	**Sham PT + Home Exercise + Text Messages**	**Standard PT + Home Exercise + Text Messages**
**Baseline**	KOOS Pain	218 (100%)	222 (100%)	220 (100%)	219 (100%)
	KOOS ADL	218 (100%)	222 (100%)	219 (99.5%)	218 (99.5%)
	EQ-5D	213 (98%)	220 (99%)	213 (97%)	215 (98%)
	Gluteal strength	208 (95%)	208 (94%)	212 (97%)	210 (96%)
	Hamstrings strength	210 (96%)	212 (95%)	213 (97%)	210 (96%)
	Quadriceps strength	210 (96%)	212 (95%)	213 (97%)	210 (96%)
**Month 3**	KOOS Pain	188 (86%)	192 (86%)	193 (88%)	189 (86%)
	KOOS ADL	188 (86%)	191 (86%)	194 (88%)	189 (86%)
	EQ-5D	181 (83%)	189 (85%)	190 (86%)	188 (86%)
	Gluteal strength	137 (63%)	154 (69%)	160 (73%)	151 (69%)
	Hamstrings strength	137 (63%)	155 (70%)	161 (73%)	151 (69%)
	Quadriceps strength	137 (63%)	155 (70%)	161 (73%)	151 (69%)
**Month 6**	KOOS Pain	184 (84%)	178 (80%)	189 (86%)	186 (85%)
	KOOS ADL	184 (84%)	178 (80%)	187 (85%)	187 (85%)
	EQ-5D	178 (82%)	173 (78%)	180 (82%)	184 (84%)
**Month 12**	KOOS Pain	174 (80%)	179 (81%)	188 (85%)	190 (87%)
	KOOS ADL	175 (80%)	178 (80%)	188 (85%)	190 (87%)
	EQ-5D	171 (78%)	174 (78%)	183 (83%)	183 (84%)

Table S10: Adjusted* differences between randomization arms in change in secondary outcome measures from baseline to three months, Multiple Imputation for Missing Data

Comparison Arms		Δ KOOS ADL for A	Δ KOOS ADL for B	Difference in Δ KOOS ADL	95% CI^
**A**	**B**				
Home Exercise	Home Exercise + Text Messages	-15.8	-15.5	-0.3	-3.0 ,2.5
Home Exercise	Standard PT + Home Exercise + Text Messages	-15.8	-18.2	2.4	-0.4 ,5.1
Home Exercise + Text Messages	Standard PT + Home Exercise + Text Messages	-15.5	-18.2	2.6	-0.1 ,5.4
Home Exercise	Sham PT + Home Exercise + Text Messages	-15.8	-18.8	3.0	0.2 ,5.8
Home Exercise + Text Messages	Sham PT + Home Exercise + Text Messages	-15.5	-18.8	3.3	0.5 ,6.0
Sham PT + Home Exercise + Text Messages	Standard PT + Home Exercise + Text Messages	-18.8	-18.2	-0.6	-3.4 ,2.1
**Comparison Arms**		**Δ EQ5-D# for A**	**Δ EQ5-D for B**	**Difference in Δ EQ5-D**	**95% CI**
**A**	**B**				
Home Exercise	Home Exercise+ Text Messages	0.09	0.10	-0.013	-0.04 ,0.01
Home Exercise	Standard PT + Home Exercise + Text Messages	0.09	0.12	-0.029	-0.06 ,-0.002
Home Exercise + Text Messages	Standard PT + Home Exercise + Text Messages	0.10	0.12	-0.016	-0.04 ,0.01
Home Exercise	Sham PT + Home Exercise+ Text Messages	0.09	0.13	-0.040	-0.07 ,-0.01
Home Exercise + Text Messages	Sham PT + Home Exercise + Text Messages	0.10	0.13	-0.026	-0.05 ,0.001

Sham PT + Home Exercise + Text Messages	Standard PT + Home Exercise + Text Messages	0.13	0.12	0.010	-0.02 ,0.04
**Comparison Arms**		**Δ Quad Strength for A**	**Δ Quad Strength for B**	**Difference in Δ Quad Strength**	**95% CI**
**A**	**B**				
Home Exercise	Home Exercise + Text Messages	6.69	6.71	0.0	-2.7 ,2.7
Home Exercise	Standard PT + Home Exercise + Text Messages	6.69	7.22	-0.5	-3.3 ,2.2
Home Exercise + Text Messages	Standard PT + Home Exercise + Text Messages	6.71	7.22	-0.5	-3.2 ,2.2
Home Exercise	Sham PT + Home Exercise + Text Messages	6.69	7.33	-0.6	-3.4 ,2.1
Home Exercise + Text Messages	Sham PT + Home Exercise + Text Messages	6.71	7.33	-0.6	-3.3 ,2.1
Sham PT + Home Exercise + Text Messages	Standard PT + Home Exercise + Text Messages	7.33	7.22	0.1	-2.6 ,2.8
**Comparison Arms**		**Δ Hamstring Strength for A**	**Δ Hamstring Strength for B**	**Difference in Δ Hamstring Strength**	**95% CI**
**A**	**B**				
Home Exercise	Home Exercise + Text Messages	4.80	3.91	0.9	-0.8 ,2.6
Home Exercise	Standard PT + Home Exercise + Text Messages	4.80	5.86	-1.1	-2.8 ,0.7
Home Exercise + Text Messages	Standard PT + Home Exercise + Text Messages	3.91	5.86	-2.0	-3.7 ,-0.2
Home Exercise	Sham PT + Home Exercise + Text Messages	4.80	4.40	0.4	-1.3 ,2.1
Home Exercise + Text Messages	Sham PT + Home Exercise + Text Messages	3.91	4.40	-0.5	-2.2 ,1.2

Sham PT + Home Exercise + Text Messages	Standard PT + Home Exercise + Text Messages	4.40	5.86	-1.5	-3.2 ,0.3
**Comparison Arms**		**Δ gluteal strength for A**	**Δ gluteal strength for B**	**Difference in Δ gluteal strength**	**95% CI**
**A**	**B**				
Home Exercise	Home Exercise+ Text Messages	5.20	5.99	-0.8	-2.7 ,1.1
Home Exercise	Standard PT + Home Exercise + Text Messages	5.20	5.89	-0.7	-2.6 ,1.2
Home Exercise + Text Messages	Standard PT + Home Exercise + Text Messages	5.99	5.89	0.1	-1.8 ,2.0
Home Exercise	Sham PT + Home Exercise + Text Messages	5.20	5.07	0.1	-1.8 ,2.0
Home Exercise + Text Messages	Sham PT + Home Exercise + Text Messages	5.99	5.07	0.9	-1.0 ,2.8
Sham PT + Home Exercise + Text Messages	Standard PT + Home Exercise + Text Messages	5.07	5.89	-0.8	-2.7 ,1.1

*Adjusted for site, baseline KL grade, baseline KOOS Pain, COVID enrollment
^Confidence intervals for secondary comparisons have not been adjusted for multiplicity and may not be used in place of hypothesis testing.
#EQ-5D analyses are exploratory

Table S11: Adjusted* differences between randomization arms in change in KOOS Pain from baseline to three months: Sensitivity Analysis for Missing Data using Missing Not at Random Multiple Imputation

	Comparison Arms		Δ KOOS Pain for A	Δ KOOS Pain for B	Difference in Δ KOOS Pain (95% CI)^
**Scenario**	**A**	**B**			
1: Missing Data shifted by 0.25 standard deviation	Home Exercise	Home Exercise + Text Messages	-16.5	-16.5	0.1 (-2.8, 2.9)
	Home Exercise	Sham PT + Home Exercise + Text Messages	-16.5	-19.8	3.3 (0.5, 6.1)
	Home Exercise	Standard PT + Home Exercise + Text Messages	-16.5	-18.8	2.4 (-0.5, 5.2)
	Home Exercise + Text Messages	Sham PT + Home Exercise + Text Messages	-16.5	-19.8	3.3 (0.4, 6.1)
	Home Exercise + Text Messages	Standard PT + Home Exercise + Text Messages	-16.5	-18.8	2.3 (-0.5, 5.1)
	Sham PT + Home Exercise+ Text Messages	Standard PT + Home Exercise + Text Messages	-19.8	-18.8	-0.9 (-3.8, 1.9)
2: Missing Data shifted by 0.5 standard deviation	Home Exercise	Home Exercise + Text Messages	-15.8	-16.0	0.3 (-2.6, 3.2)
	Home Exercise	Sham PT + Home Exercise + Text Messages	-15.8	-19.2	3.5 (0.6, 6.4)
	Home Exercise	Standard PT + Home Exercise + Text Messages	-15.8	-18.1	2.4 (-0.5, 5.3)
	Home Exercise + Text Messages	Sham PT + Home Exercise + Text Messages	-16.0	-19.2	3.2 (0.3, 6.1)
	Home Exercise + Text Messages	Standard PT + Home Exercise + Text Messages	-16.0	-18.1	2.1 (-0.8, 5.0)
	Sham PT + Home Exercise + Text Messages	Standard PT + Home Exercise + Text Messages	-19.2	-18.1	-1.1 (-4.0, 1.8)
3: Missing Data shifted by 1 standard deviation	Home Exercise	Home Exercise + Text Messages	-14.8	-15.0	0.2 (-2.8, 3.2)
	Home Exercise	Sham PT + Home Exercise + Text Messages	-14.8	-18.2	3.4 (0.4, 6.5)
	Home Exercise	Standard PT + Home Exercise + Text Messages	-14.8	-17.1	2.3 (-0.7, 5.4)
	Home Exercise + Text Messages	Sham PT + Home Exercise + Text Messages	-15.0	-18.2	3.3 (0.2, 6.3)

	Home Exercise + Text Messages	Standard PT + Home Exercise + Text Messages	-15.0	-17.1	2.2 (-0.9, 5.2)
	Sham PT + Home Exercise + Text Messages	Standard PT + Home Exercise + Text Messages	-18.2	-17.2	-1.1 (-4.1, 1.9)
4: Missing Data shifted by 0.5 standard deviation for arms 1 and 2 only	Home Exercise	Home Exercise + Text Messages	-15.6	-15.9	0.3 (-2.5, 3.2)
	Home Exercise	Sham PT + Home Exercise + Text Messages	-15.6	-20.1	4.4 (1.6, 7.3)
	Home Exercise	Standard PT + Home Exercise + Text Messages	-15.6	-19.2	3.6 (0.8, 6.5)
	Home Exercise + Text Messages	Sham PT + Home Exercise + Text Messages	-15.9	-20.1	4.1 (1.3, 7.0)
	Home Exercise + Text Messages	Standard PT + Home Exercise + Text Messages	-15.9	-19.2	3.3 (0.5, 6.2)
	Sham PT + Home Exercise + Text Messages	Standard PT + Home Exercise + Text Messages	-20.1	-19.2	-0.8 (-3.7, 2.0)
5: Missing Data shifted by 0.5 standard deviation for arms 3 and 4 only	Home Exercise	Home Exercise + Text Messages	-17.2	-17.1	-0.1 (-3.1, 2.9)
	Home Exercise	Sham PT + Home Exercise + Text Messages	-17.2	-18.0	0.8 (-2.1, 3.8)
	Home Exercise	Standard PT + Home Exercise + Text Messages	-17.2	-16.8	-0.4 (-3.4, 2.6)
	Home Exercise + Text Messages	Sham PT + Home Exercise + Text Messages	-17.1	-18.0	0.9 (-2.0, 3.9)
	Home Exercise + Text Messages	Standard PT + Home Exercise + Text Messages	-17.1	-16.8	-0.3 (-3.3, 2.7)
	Sham PT + Home Exercise + Text Messages	Standard PT + Home Exercise + Text Messages	-18.0	-16.8	-1.2 (-4.2, 1.7)

^Confidence intervals have not been adjusted for multiplicity and may not be used in place of hypothesis testing

Table S12: Primary and Secondary 3-Month Outcomes by Site													
		**Boston**			**Buffalo**			**Cleveland**			**Pittsburgh**		
**Outcome**	**Month**	**N**	**Mean**	**Median**	**N**	**Mean**	**Median**	**N**	**Mean**	**Median**	**N**	**Mean**	**Median**
KOOS Pain	0	269	45.0	44.4	450	46.8	47.2	73	46.9	44.4	87	44.6	44.4
	3	233	28.6	25.0	398	26.5	25.0	56	27.6	22.2	75	24.6	22.2
	6	221	26.2	22.2	386	23.7	19.4	57	22.6	19.4	73	22.5	19.4
	12	223	22.1	19.4	382	20.1	16.7	53	19.3	16.7	73	20.1	16.7
KOOS ADL	0	268	34.4	32.5	449	36.7	37.5	73	36.4	35.0	87	30.6	32.5
	3	232	20.1	15.0	401	17.6	12.5	55	15.4	7.5	74	13.9	10.0
	6	221	18.3	10.0	385	16.4	10.0	57	13.3	7.5	73	12.6	7.5
	12	224	16.0	8.9	382	13.9	7.5	52	11.9	7.5	73	12.2	7.5
EQ-5D	0	263	0.687	0.719	440	0.677	0.712	72	0.688	0.709	86	0.698	0.738
	3	227	0.771	0.787	394	0.795	0.826	54	0.824	0.878	73	0.840	0.872
	6	212	0.792	0.844	374	0.828	0.872	57	0.830	0.883	72	0.849	0.878
	12	216	0.811	0.872	373	0.839	0.875	52	0.882	0.940	70	0.860	0.891
Quad Strength	0	247	42.3	38.5	445	49.7	46.2	66	51.7	44.1	87	68.6	63.3
	3	180	49.6	44.2	337	58.3	53.6	26	59.5	53.4	61	74.8	68.1
Ham-string Strength	0	247	24.2	22.9	445	31.4	29.2	66	30.9	29.7	87	35.2	32.9
	3	180	28.4	26.4	337	38.1	35.2	26	32.6	30.9	61	40.2	37.1
Gluteal strength	0	243	28.8	26.7	443	35.9	34.1	65	41.8	39.5	87	45.4	44.8
	3	179	33.9	31.2	336	42.2	39.3	26	47.6	45.1	61	50.6	48.5

Table S13: Adjusted* 3-Month Outcomes by Site and Randomization Arm.					
		Adjusted 3-Month Change from Baseline (95% CI)^			
**Outcome**	**Treatment Arm**	**Boston**	**Buffalo**	**Cleveland**	**Pittsburgh**
KOOS Pain	Home Exercise	-15.4 (-19.2, -11.6)	-17.7 (-20.6, -14.8)	-19.6 (-28.9, -10.3)	-20.5 (-27.4, -13.6)
	Home Exercise + Text Messages	-12.4 (-16.2, -8.5)	-19.9 (-22.8, -16.9)	-19.1 (-26.4, -11.7)	-16.4 (-23.0, -9.8)
	Sham PT + Home Exercise + Text Messages	-19.7 (-23.5, -15.8)	-20.3 (-23.3, -17.4)	-14.8 (-22.4, -7.2)	-26.3 (-33.2, -19.3)
	Standard PT + Home Exercise + Text Messages	-19.5 (-23.4, -15.6)	-19.9 (-22.9, -17.0)	-19.0 (-26.6, -11.4)	-18.8 (-25.5, -12.0)
KOOS ADL	Home Exercise	-11.8 (-15.6, -8.0)	-16.8 (-19.7, -14.0)	-18.7 (-27.9, -9.5)	-16.9 (-23.7, -10.1)
	Home Exercise + Text Messages	-9.9 (-13.7, -6.0)	-17.7 (-20.7, -14.8)	-16.6 (-24.0, -9.1)	-14.5 (-21.2, -7.8)
	Sham PT + Home Exercise + Text Messages	-16.4 (-20.2, -12.7)	-18.6 (-21.5, -15.7)	-19.1 (-26.6, -11.7)	-21.3 (-28.1, -14.5)
	Standard PT + Home Exercise + Text Messages	-17.6 (-21.4, -13.7)	-16.6 (-19.6, -13.7)	-19.2 (-26.7, -11.7)	-18.8 (-25.5, -12.2)
EQ-5D#	Home Exercise	0.032 (-0.008, 0.07)	0.097 (0.068, 0.127)	0.109 (0.013, 0.206)	0.102 (0.032, 0.172)
	Home Exercise + Text Messages	0.067 (0.029, 0.106)	0.101 (0.071, 0.130)	0.116 (0.041, 0.190)	0.105 (0.038, 0.172)
	Sham PT + Home Exercise + Text Messages	0.101 (0.063, 0.140)	0.116 (0.086, 0.145)	0.114 (0.039, 0.188)	0.152 (0.082, 0.222)
	Standard PT + Home Exercise + Text Messages	0.098 (0.059, 0.136)	0.101 (0.071, 0.131)	0.107 (0.029, 0.185)	0.176 (0.109, 0.242)
Quad Strength	Home Exercise	6.9 (2.2, 11.6)	7.7 (4.6, 10.8)	7.2 (-6.7, 21.0)	6.4 (-0.8, 13.6)
	Home Exercise + Text Messages	7.5 (3.3, 11.8)	7.4 (4.4, 10.4)	5.8 (-5.6, 17.1)	4.6 (-2.7, 11.8)
	Sham PT + Home Exercise + Text Messages	3.9 (0.0, 7.9)	9.1 (6.1, 12.1)	15.7 (5.8, 25.5)	6.0 (-0.8, 12.8)
	Standard PT + Home Exercise + Text Messages	5.5 (1.3, 9.7)	7.6 (4.5, 10.7)	7.5 (-2.4, 17.3)	9.9 (2.5, 17.3)

**Table S13: Adjusted* 3-Month Outcomes by Site and Randomization Arm.**					
		**Adjusted 3-Month Change from Baseline (95% CI)^**			
**Outcome**	**Treatment Arm**	**Boston**	**Buffalo**	**Cleveland**	**Pittsburgh**
Hamstring strength	Home Exercise	2.1<br>(-0.9, 5.1)	7.2<br>(5.3, 9.2)	0.9<br>(-7.7, 9.6)	5.2<br>(0.7, 9.7)
	Home Exercise + Text Messages	1.6<br>(-1.1, 4.2)	6.1<br>(4.2, 7.9)	-0.8<br>(-7.9, 6.2)	4.4<br>(-0.1, 8.9)
	Sham PT + Home Exercise + Text Messages	1.2<br>(-1.3, 3.7)	6.9<br>(5.0, 8.7)	-0.8<br>(-7.0, 5.3)	6.1<br>(1.9, 10.3)
	Standard PT + Home Exercise + Text Messages	5.1<br>(2.5, 7.8)	6.6<br>(4.7, 8.5)	1.0<br>(-5.1, 7.1)	5.3<br>(0.6, 9.9)
Gluteal strength	Home Exercise	1.3<br>(-1.9, 4.6)	5.8<br>(3.7, 8.0)	2.2<br>(-7.4, 11.8)	7.9<br>(2.9, 12.9)
	Home Exercise + Text Messages	3.3<br>(0.2, 6.3)	6.5<br>(4.4, 8.6)	7.0<br>(-0.9, 14.8)	7.5<br>(2.5, 12.5)
	Sham PT + Home Exercise + Text Messages	2.9<br>(0.1, 5.6)	5.8<br>(3.8, 7.9)	8.5<br>(1.7, 15.3)	3.5<br>(-1.2, 8.1)
	Standard PT + Home Exercise + Text Messages	4.1<br>(1.2, 7.0)	5.3<br>(3.2, 7.4)	3.4<br>(-3.4, 10.3)	6.0<br>(0.9, 11.2)

*****Adjusted for site, baseline KL grade, baseline KOOS Pain, COVID enrollment in multivariable linear regression;

^Confidence intervals for secondary comparisons have not been adjusted for multiplicity and may not be used in place of hypothesis testing.

[#]EQ-5D analyses are exploratory

*Adjusted for site, baseline KOOS Pain, COVID enrollment in multivariable linear regression

Table S14: Adjusted* 3-Month Outcomes by Site and KL Grade (0-1 vs. 2-3)

		Adjusted 3-Month Change from Baseline (95% CI)^	
**Outcome**	**Trt Arm**	**KLG 0-1**	**KLG 2-3**
KOOS Pain	Home Exercise	-17.0 (-20.0, -14.0)	-17.9 (-21.4, -14.4)
	Home Exercise + Text Messages	-18.9 (-22.2, -15.7)	-15.5 (-18.6, -12.4)
	Sham PT + Home Exercise + Text Messages	-20.3 (-23.3, -17.3)	-20.4 (-23.7, -17.0)
	Standard PT + Home Exercise+ Text Messages	-19.1 (-22.2, -16.0)	-20.2 (-23.5, -17.0)
KOOS ADL	Home Exercise	-16.6 (-19.6, -13.6)	-14.9 (-18.3, -11.4)
	Home Exercise + Text Messages	-17.9 (-21.1, -14.7)	-12.9 (-15.9, -9.8)
	Sham PT+ Home Exercise + Text Messages	-18.8 (-21.8, -15.9)	-18.5 (-21.8, -15.2)
	Standard PT+ Home Exercise + Text Messages	-17.9 (-21.0, -14.9)	-17.5 (-20.7, -14.3)
EQ-5D#	Home Exercise	0.090 (0.059, 0.121)	0.080 (0.044, 0.115)
	Home Exercise + Text Messages	0.124 (0.092, 0.156)	0.075 (0.044, 0.105)
	Sham PT+ Home Exercise + Text Messages	0.122 (0.092, 0.152)	0.121 (0.088, 0.155)
	Standard PT+ Home Exercise + Text Messages	0.102 (0.071, 0.132)	0.131 (0.098, 0.164)
Quad Strength	Home Exercise	7.7 (4.2, 11.2)	7.2 (3.3, 11.1)
	Home Exercise + Text Messages	8.4 (4.8, 12.0)	6.0 (2.5, 9.5)
	Sham PT+ Home Exercise + Text Messages	7.6 (4.3, 10.9)	7.9 (4.4, 11.4)
	Standard PT+ Home Exercise + Text Messages	7.4 (3.9, 11.0)	7.2 (3.7, 10.7)
Hamstring strength	Home Exercise	4.3 (2.1, 6.5)	3.2 (0.8, 5.7)
	Home Exercise + Text Messages	3.9 (1.7, 6.1)	1.8 (-0.4, 4.0)
	Sham PT+ Home Exercise + Text Messages	3.9 (1.9, 5.9)	2.7 (0.5, 4.9)
	Standard PT+ Home Exercise+ Text Messages	3.9 (1.7, 6.1)	5.0 (2.8, 7.1)
Gluteal strength	Home Exercise	5.1 (2.7, 7.6)	4.3 (1.6, 7.0)
	Home Exercise + Text Messages	6.8 (4.3, 9.3)	4.7 (2.3, 7.2)

Table S14: Adjusted* 3-Month Outcomes by Site and KL Grade (0-1 vs. 2-3)			
Adjusted 3-Month Change from Baseline (95% CI)^			
**Outcome**	**Trt Arm**	**KLG 0-1**	**KLG 2-3**
	Sham PT+ Home Exercise + Text Messages	4.8 (2.5, 7.0)	5.2 (2.7, 7.6)
	Standard PT+ Home Exercise + Text Messages	4.6 (2.1, 7.0)	5.5 (3.1, 7.9)

*Adjusted for site, baseline KL grade, baseline KOOS Pain, COVID enrollment in multivariable linear regression;

^Confidence intervals for secondary comparisons have not been adjusted for multiplicity and may not be used in place of hypothesis testing.

#EQ-5D analyses are exploratory

Table S15: Adverse events in TeMPO Trial by Randomization Arm (Event Level)

AE and SAE	Entire Cohort	Home Exercise	Home Exercise + Text Messages	Sham PT + Home Exercise + Text Messages	Standard PT + Home Exercise + Text Messages
Arthroscopic Partial Meniscectomy in index knee	80	21	21	18	20
Disabling* pain in index knee	55	17	16	12	10
Emergency Department visit					
Cardiovascular	9	1	3	4	1
Neurological	5	2	1	1	1
Pulmonary	4	1	0	3	0
Infectious	6	4	0	0	2
Other	87	28	23	26	10
Adverse Event: Total	246	74	64	64	44
Death	1	0	0	0	1
Hospitalization	38	15	10	6	7
Serious Adverse Event: Total	39	15	10	6	8

*Causing subject to be unable to walk, or unable to walk without an assistive device, for at least one day

Supplement References

1. Roos EM, Roos HP, Lohmander LS, Ekdahl C, Beynnon BD. Knee Injury and Osteoarthritis Outcome Score (KOOS)—Development of a Self-Administered Outcome Measure. Journal of Orthopaedic & Sports Physical Therapy 1998;28:88-96.
2. Fransen M, Edmonds J. Reliability and validity of the EuroQol in patients with osteoarthritis of the knee. Rheumatology (Oxford) 1999;38:807-13.
3. Holm PM, Nyberg M, Wernbom M, Schroder HM, Skou ST. Intrarater Reliability and Agreement of Recommended Performance-Based Tests and Common Muscle Function Tests in Knee Osteoarthritis. J Geriatr Phys Ther 2021;44:144-52.
4. Dobson F, Hinman RS, Hall M, et al. Reliability and measurement error of the Osteoarthritis Research Society International (OARSI) recommended performance-based tests of physical function in people with hip and knee osteoarthritis. Osteoarthritis Cartilage 2017;25:1792-6.
